# Supplementary material for: Mapping the Genomic Regions Controlling Germination Rate and Early Seedling Growth Parameters in Rice
Source: Genes (Basel). 2023 Apr 12;14(4):902. doi: 10.3390/genes14040902 (PMC10138111; doi:10.3390/genes14040902)
Supplement: Supplementary file 1 [file genes-14-00902-s001.zip › genes-2206484-supplementary.pdf]

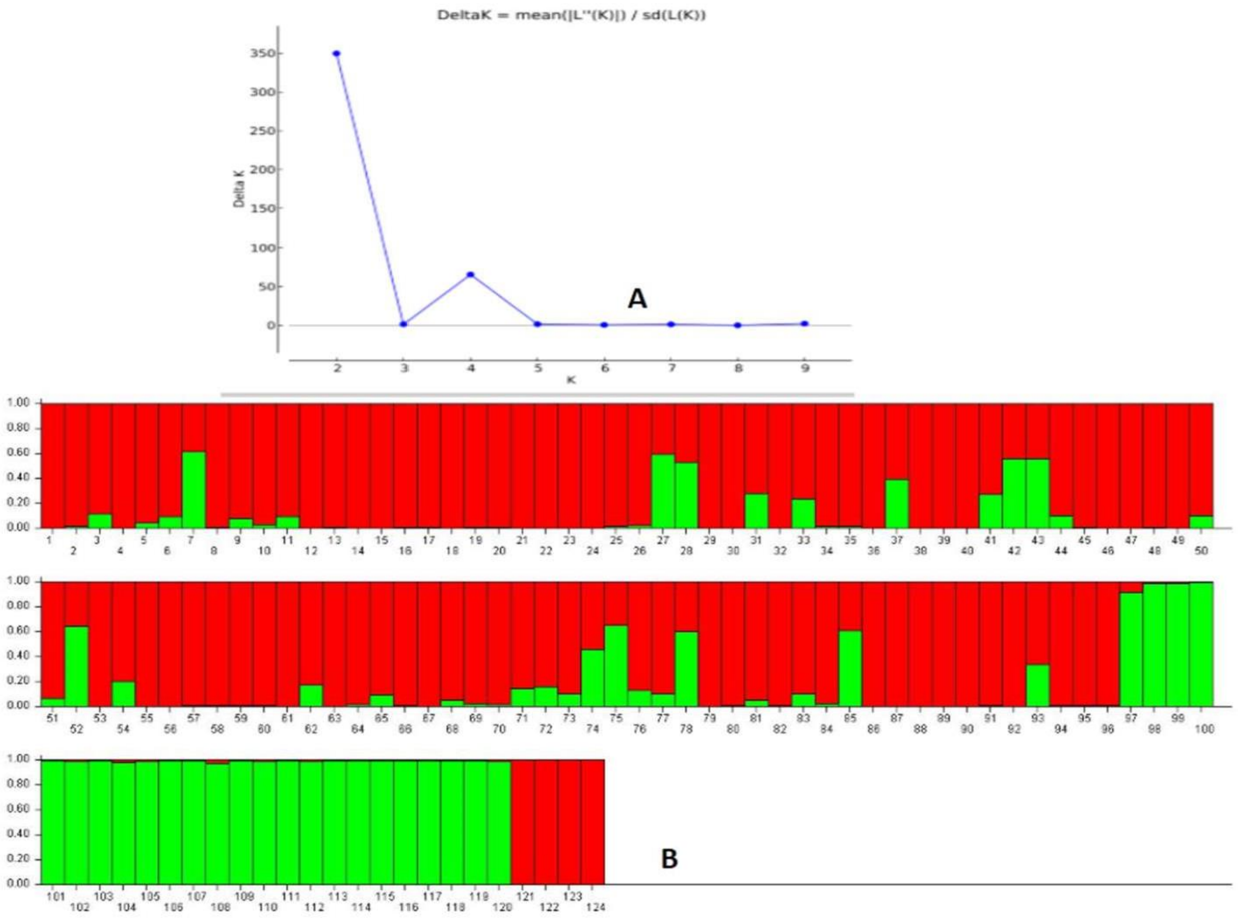

Supplementary Figure S1. (A) Graph of ΔK value, to the rate of change in the log probability of data between successive K values; (**B)** Population structure of the 108 panel population based on membership probability fractions of individual genotypes at K = 2. The genotypes with the probability of ≥80% membership proportions were assigned as subgroups while others grouped as admixture group. The numbers in the diagram depict the serial number of the germplasm lines listed in Table 1.

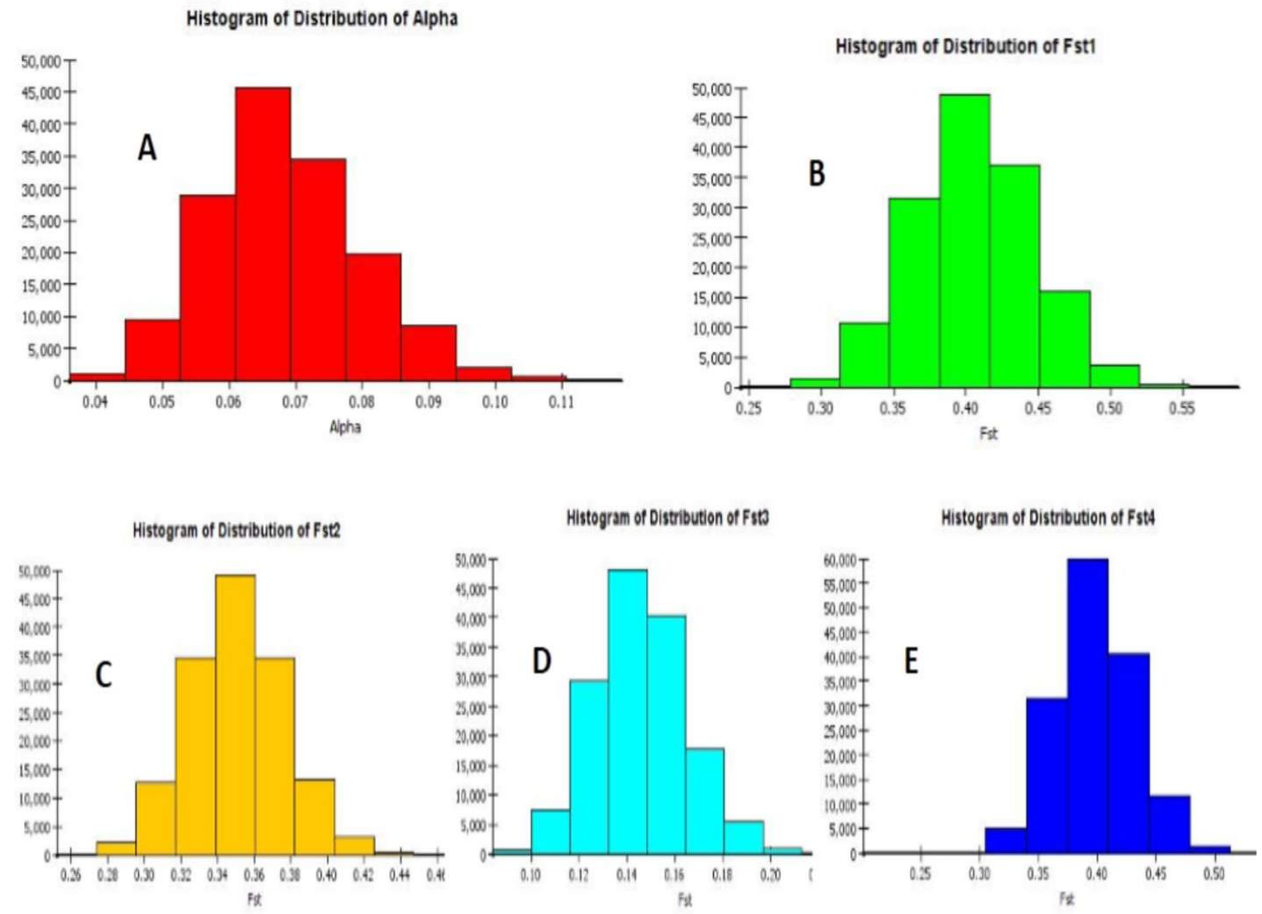

Supplementary Figure S2. The distribution pattern of alpha and F_{ST} values (A) Alpha value of the population at K = 4 and (B–E) four subpopulations at K = 4 showing a symmetric shape of the four Fst values.

Supplementary Table 1. Mean estimates of germination rate and early seedling growth parameters estimated from 278 rice germlasm lines.

Sl. No.	Name of the germplasm	RSG	RGR	AGR	MGR
1	*Magura-s	1.380	0.241	1.857	0.181
2	Maguramanji	1.173	0.058	1.657	0.228
3	Kalakanhu	1.245	0.062	0.785	0.232
4	*Magra	0.846	0.175	2.065	0.204
5	Jata	0.785	0.154	0.845	0.325
6	*Kathidhan	0.436	0.091	0.871	0.189
7	Gargarh	0.982	0.113	1.121	0.156
8	Jhuli	1.112	0.089	0.725	0.245
9	*Karpurkanti	0.694	0.073	1.004	0.211
10	Rasuna	0.845	0.112	0.645	0.169

11	*Lalgundi	0.584	0.137	0.841	0.219
12	Latasal	0.781	0.071	1.105	0.258
13	Kasibaha	0.689	0.074	0.746	0.172
14	*Latamahu	0.317	0.212	1.149	0.194
15	Rangi	0.941	0.087	0.658	0.241
16	Kalakadam	0.853	0.078	0.696	0.186
17	*Kantakaamala	0.410	0.085	1.114	0.213
18	Pankhi	0.912	0.112	0.721	0.211
19	*Kanakchampa	0.448	0.301	1.107	0.182
20	Sodi	0.877	0.089	0.845	0.193
21	*Kundadhan	0.764	0.238	0.928	0.165
22	Bhundi	0.763	0.187	0.984	0.218
23	*Landi	0.614	0.151	1.224	0.207
24	Daonra	0.985	0.115	1.112	0.214
25	*kapanthi	1.463	0.071	2.170	0.219
26	Pasibutia	0.883	0.143	1.018	0.211
27	*LatAc.haunri	0.246	0.196	0.658	0.218
28	Salkoiya	0.785	0.158	1.142	0.208
29	*Laxmibilash	0.382	0.261	0.650	0.196
30	Guntha	0.825	0.146	1.136	0.235
31	Malata	0.863	0.089	0.987	0.189
32	*Kantakapura	0.975	0.116	1.385	0.231
33	*Chinamal	0.528	0.186	1.015	0.208
34	Pindagadi	0.998	0.097	0.728	0.169
35	Baunsagaja	0.946	0.102	0.984	0.173
36	*GondiAc.hampeisiali	0.870	0.089	1.325	0.217
37	Palasphul	0.754	0.113	0.993	0.208
38	*BalisaralaktimAc.hi -k	0.968	0.136	2.080	0.231
39	Matia	0.789	0.109	1.112	0.211
40	Sarada	0.863	0.124	1.108	0.162
41	*Champaeisiali	0.936	0.110	2.058	0.231
42	Ladu	0.987	0.162	1.119	0.185
43	*Kaniar	0.843	0.051	1.250	0.225
44	Madhabi	0.962	0.175	0.987	0.167
45	AC.9011	1.279	0.116	2.100	0.268
46	*AC.9050	0.254	0.148	0.469	0.258
47	AC.9102	0.647	0.294	1.383	0.231
48	AC.9119A	0.310	0.227	0.653	0.214
49	*AC.9093	0.564	0.162	0.996	0.302
50	AC.9008	0.658	0.112	0.854	0.245
51	AC.9010	0.525	0.215	0.754	0.213

52	*AC.9043	1.286	0.023	1.536	0.313
53	AC.9016	0.463	0.186	0.689	0.308
54	AC.9019	0.786	0.245	1.154	0.256
55	*AC.9044A	0.986	0.268	2.285	0.280
56	AC.9022	0.742	0.224	0.894	0.287
57	AC.9023	0.683	0.124	0.879	0.243
58	AC.9025	0.712	0.185	0.785	0.215
59	*AC.9005	1.121	0.098	1.875	0.302
60	AC.9059	0.652	0.162	0.776	0.325
61	*AC.9076A	0.139	0.199	0.681	0.258
62	AC.9063	0.732	0.223	0.721	0.346
63	AC.9076	0.652	0.174	0.658	0.328
64	*AC.9030	1.665	0.243	2.285	0.272
65	AC.9081	0.559	0.165	0.842	0.214
66	*AC.9058	1.126	0.123	1.428	0.278
67	AC.9086	0.694	0.189	0.814	0.258
68	AC.9095	0.712	0.214	0.786	0.223
69	*AC.9028	1.365	0.119	2.302	0.311
70	AC.9097	0.652	0.225	0.774	0.215
71	*AC.9065	0.869	0.128	1.196	0.325
72	AC.9099	0.559	0.189	0.658	0.219
73	AC.9118	0.638	0.178	0.846	0.223
74	*AC.9063	1.121	0.131	1.597	0.286
75	AC.9155	0.611	0.162	0.723	0.224
76	AC.9157	0.584	0.149	0.645	0.221
77	AC.9232	1.121	0.115	1.124	0.251
78	*AC.9006	1.189	0.123	1.896	0.292
79	AC.9241	1.541	0.164	1.152	0.241
80	*AC.9053A	0.857	0.013	0.997	0.235
81	AC.9248	1.329	0.148	1.128	0.218
82	AC.9268	1.116	0.148	0.887	0.278
83	*AC.9021	1.113	0.151	1.412	0.282
84	AC.9273	0.897	0.164	0.985	0.235
85	*AC.9038	1.638	0.281	2.543	0.281
86	AC.9278	0.984	0.158	0.846	0.264
87	AC.9077	0.998	0.089	1.114	0.283
88	AC.9094	1.165	0.092	1.105	0.234
89	AC.9096	1.143	0.098	0.785	0.225
90	*AC.9035	1.754	0.118	2.657	0.295
91	AC.9122	1.542	0.141	0.798	0.215
92	*AC.9090	0.812	0.165	1.254	0.292

93	AC.9127	1.878	0.164	0.845	0.222
94	*Pk-21	1.597	0.061	2.387	0.235
95	*Mahamaga	0.621	0.105	1.113	0.223
96	Umamata	0.930	0.180	1.470	0.207
97	* D1	0.488	0.245	0.863	0.203
98	Uma	0.359	0.105	0.658	0.157
99	M-10	0.451	0.115	0.645	0.213
100	*Ezhoml-2	0.668	0.261	0.942	0.225
101	Nambial Amber	0.325	0.165	0.556	0.195
102	Aiswarya	0.463	0.215	0.821	0.214
103	*Kozhivalan	0.532	0.216	0.630	0.228
104	Kanchan	0.514	0.189	0.658	0.152
105	*Cheruvirippu	0.516	0.089	0.808	0.231
106	KunjeKunje	0.328	0.225	0.725	0.116
107	Harsha	0.412	0.185	0.625	0.227
108	*Adira-3 Pallakad	0.846	0.112	1.345	0.232
109	Kanjana	0.369	0.235	0.821	0.185
110	Adhuthurai	0.358	0.242	0.632	0.226
111	*Gandhakasala	0.690	0.102	1.412	0.231
112	Ambeponar	0.459	0.165	0.725	0.163
113	* Sreyas	0.711	0.071	1.358	0.225
114	*Jaya	0.511	0.279	1.011	0.228
115	Triveni	0.389	0.342	1.235	0.224
116	*PK6	0.635	0.165	1.079	0.211
117	Chengarama	0.412	0.258	1.212	0.221
118	*Adira-1 Pallakad	0.891	0.071	1.121	0.236
119	*Jyothi	0.732	0.256	1.146	0.211
120	Kuttosam	0.383	0.126	0.825	0.156
121	*CR4034-77-5	0.692	0.173	0.825	0.189
122	*CR4034-77-2	0.725	0.181	0.912	0.178
123	*Jalamagna	0.930	0.114	2.320	0.220
124	*Panidhan	0.900	0.081	1.390	0.201
125	Thavalakkaman	0.396	0.154	0.875	0.163
126	*Marathondi	1.075	0.061	1.329	0.231
127	Champanmata	0.423	0.187	0.965	0.187
128	Kalladiyaran	0.408	0.196	0.745	0.176
129	*Adira-2 Pallakad	0.851	0.073	1.632	0.238
130	Onam	0.374	0.214	0.758	0.189
131	*VAc.haw	0.242	0.115	0.633	0.236
132	Nooravella	0.368	0.211	0.942	0.195
133	Airweraga	0.586	0.133	0.836	0.221

134	Chetadi	0.411	0.217	0.932	0.184
135	Amballavalaya	0.453	0.231	0.914	0.211
136	AC.20117	0.584	0.112	0.847	0.232
137	*AC.20770	1.209	0.052	1.512	0.248
138	AC.20436	1.090	0.170	2.063	0.231
139	*AC.20423	0.612	0.149	1.712	0.218
140	AC.20604	0.940	0.141	1.937	0.244
141	*AC.20614	0.199	0.153	0.519	0.241
142	*AC.20686	0.208	0.162	0.374	0.256
143	AC.20690	0.643	0.107	0.890	0.248
144	*AC.20845	1.081	0.112	1.349	0.231
145	*AC.20627	0.705	0.014	0.928	0.248
146	AC.20071	0.687	0.185	0.857	0.214
147	*AC.20664	0.293	0.084	0.519	0.236
148	*AC.20907	0.528	0.069	0.812	0.228
149	AC.20081	0.745	0.151	0.754	0.205
150	*AC.20246	0.876	0.145	1.278	0.271
151	AC.20095	0.732	0.164	0.658	0.218
152	AC.20113	0.685	0.089	0.712	0.234
153	*AC.20282	1.192	0.105	2.019	0.258
154	*AC.20328	1.085	0.105	1.589	0.287
155	AC.20119	0.657	0.104	1.125	0.189
156	*AC.20371	1.153	0.107	1.487	0.301
157	AC.20121	0.711	0.164	1.321	0.192
158	*AC.20347	0.687	0.150	1.248	0.218
159	AC.20128	0.987	0.152	0.845	0.211
160	*AC.20362	0.782	0.100	1.179	0.293
161	*AC.20389	0.711	0.112	1.114	0.275
162	AC.20136	0.841	0.147	0.741	0.208
163	*AC.20317	0.172	0.148	0.452	0.291
164	*AC.20920	0.935	0.043	2.081	0.223
165	AC.20137	0.743	0.138	0.785	0.217
166	AC.5946	0.520	0.168	0.845	0.165
167	*AC.10187	0.672	0.159	2.587	0.210
168	AC.6006	0.570	0.130	1.393	0.187
169	*AC.6023	0.958	0.128	2.145	0.197
170	AC.6617	0.940	0.362	1.573	0.182
171	*AC.7008	0.725	0.196	1.423	0.186
172	AC.7009	0.530	0.088	1.093	0.187
173	AC.7124	0.373	0.153	0.663	0.182
174	AC.7204	0.763	0.314	1.420	0.182

175	*AC.7269	0.668	0.251	1.243	0.191
176	AC.10333	0.630	0.137	1.007	0.174
177	AC.10438	0.310	0.218	0.807	0.196
178	*AC.10608	0.850	0.121	1.290	0.200
179	AC.5757	0.445	0.185	1.142	0.184
180	AC.5768	0.352	0.214	1.365	0.175
181	AC.5813	0.412	0.165	1.254	0.168
182	AC.5828	0.425	0.321	1.157	0.205
183	AC.5832	0.456	0.221	0.987	0.165
184	AC.5840	0.440	0.135	0.864	0.154
185	*AC.6183	0.540	0.193	2.143	0.227
186	AC.5951	0.412	0.176	0.758	0.132
187	AC.5965	0.520	0.211	0.897	0.154
188	*AC.6027	1.021	0.249	1.632	0.193
189	*AC.10162	0.925	0.087	1.996	0.196
190	AC.6148	0.510	0.185	1.114	0.178
191	*AC.7282	0.986	0.083	1.225	0.189
192	AC.6156	0.480	0.221	1.125	0.162
193	AC.6235	0.470	0.185	0.875	0.158
194	*AC.6221	0.669	0.320	1.279	0.213
195	AC.6237	0.520	0.265	0.985	0.143
196	AC.6562	0.450	0.145	0.985	0.189
197	AC.6571	0.510	0.157	1.124	0.171
198	*AC.-5993	0.312	0.352	0.568	0.186
199	AC.6633	0.480	0.152	1.145	0.135
200	*AC.7134	0.679	0.227	1.293	0.189
201	AC.7031	0.410	0.148	1.045	0.138
202	AC.7041	0.470	0.176	0.984	0.149
203	*AC.6170	0.582	0.108	1.018	0.209
204	AC.7073	0.480	0.138	0.853	0.162
205	*AC.6007	0.884	0.096	1.752	0.188
206	AC.7089	0.510	0.149	1.237	0.178
207	AC.7093	0.420	0.224	0.995	0.194
208	*AC.6172	0.448	0.272	1.035	0.219
209	AC.7188	0.400	0.157	0.869	0.208
210	AC. 7135	0.450	0.142	0.794	0.114
211	AC. 7147	0.420	0.138	0.852	0.138
212	Polinadhan 2	0.626	0.159	0.816	0.218
213	Gerwathor	0.693	0.163	1.390	0.232
214	*Uttarbanga local-9	0.112	0.169	0.321	0.243
215	Rohidhan-1	0.880	0.217	1.057	0.218

216	*Joha	0.290	0.189	0.512	0.213
217	Manaharrathori	0.430	0.233	0.753	0.212
218	Ganrohibuna	0.580	0.183	0.723	0.250
219	Kadamful	0.540	0.183	1.243	0.236
220	*Jhingesal	0.942	0.189	1.552	0.249
221	Bakuldhan	0.173	0.046	0.650	0.268
222	*Gochi	0.447	0.159	0.697	0.246
223	Kaikee	0.727	0.241	1.140	0.250
224	*Palina dhan-1	0.659	0.081	0.931	0.241
225	Bathidhan	0.345	0.189	1.256	0.221
226	Kauka	0.542	0.221	0.956	0.238
227	Param nada	0.446	0.154	0.845	0.256
228	Sitaluchi	0.441	0.196	1.121	0.185
229	*Shayam	0.479	0.073	1.245	0.286
230	Kukurjali	0.384	0.253	0.856	0.197
231	Durudhan	0.376	0.146	0.997	0.212
232	Khara	0.449	0.165	1.243	0.254
233	Hetomari	0.521	0.194	0.987	0.232
234	*Dad ghani	0.360	0.098	0.487	0.263
235	Khajurchar	0.436	0.218	1.658	0.215
236	Pawandhan	0.385	0.225	2.145	0.249
237	Meghjawain	0.512	0.165	1.352	0.182
238	*Basumati-B	0.673	0.069	0.946	0.263
239	Bharati	0.469	0.235	1.475	0.149
240	*Chatuimuchi	0.512	0.137	1.148	0.261
241	Seshphal	0.327	0.186	1.698	0.164
242	Kaalibank	0.365	0.221	0.987	0.213
243	kaloghandeswari	0.258	0.089	0.874	0.185
244	Jaldhapa	0.247	0.211	0.965	0.192
245	*Jhagrikartik	0.327	0.104	1.257	0.226
246	Chingrifuli	0.312	0.205	0.771	0.164
247	Lalkadhan	0.249	0.185	0.845	0.208
248	*Bharati	0.736	0.179	0.864	0.235
249	Moriadhan	0.225	0.198	0.961	0.149
250	Kashiyabinni	0.214	0.148	1.102	0.168
251	Kotki	0.278	0.151	0.995	0.198
252	*Sugandha-2	0.429	0.033	0.920	0.279
253	Domnadhan	0.158	0.128	0.976	0.208
254	Kauka	0.214	0.149	1.114	0.211
255	AC. 44585	0.750	0.064	0.611	0.333
256	AC. 44588	1.900	0.023	0.717	0.333

257	AC. 44591	3.100	0.025	1.144	0.333
258	AC. 44592	1.517	0.047	0.883	0.333
259	AC. 44594	5.100	0.045	2.150	0.333
260	AC. 44595	2.283	0.023	0.856	0.333
261	AC. 44597	3.583	0.023	1.594	0.333
262	AC. 44598	0.200	0.056	0.500	0.333
263	AC. 44638	3.433	0.030	2.006	0.333
264	AC. 44646	2.033	0.033	1.200	0.333
265	AC. 44603	2.050	0.044	1.100	0.333
266	AC. 44604	1.717	0.037	0.622	0.333
267	AC. 43658	4.850	0.027	2.617	0.293
268	AC.43660	8.983	0.101	4.450	0.310
269	AC.43661	3.683	0.051	2.089	0.323
270	AC.43662	4.433	0.034	2.356	0.327
271	AC.43663	3.467	0.032	1.556	0.301
272	AC.43669	6.533	0.038	3.722	0.325
273	AC.43670	2.483	0.075	1.689	0.417
274	AC.43675	2.517	0.050	1.633	0.293
275	AC.43676	4.567	0.034	2.250	0.290
276	AC.43732	4.333	0.091	2.294	0.296
277	AC. 43737	6.650	0.054	3.828	0.310
278	AC. 43738	0.067	0.047	0.756	0.286
CV %		12.342	13.121	12.053	5.351
$LSD_{5\%}$		0.207	0.034	0.295	0.095

Supplementary Table S2. Simple sequence repeat markers used for association mapping of germination rate and early seedling growth parameters in a panel population of 124 rice germplasm lines.

SL No	PR Name	Ch No	Position (bp)	Forward	Reverse	Repeat motif	AT
1	**RM5310**	1	41197253	TAGACAAAGCAACGGGTTCC	CGGAAGCAGGAGAATCGTAG	(TC)12	55
2	**RM582**	1	9190478	TCTGTTGCCGATTTGTTCG	AAATGGCTTACCTGCTGTCTC	(TC)20	55
3	**RM13335**	2	19213952	TATGCCAAGAGGAATCCTGAAGC	GCACTCACACTGATCTGGACAGG	(GT)10	55
4	**RM6275**	2	7273421	CACTGAGCCCTTTTGTCCTC	TCCCAGATCAGAATCGAAGG	(CTG)8	50
5	**RM50**	6	6300000	ACTGTACCGGTCGAAGACG	AAATTCCACGTCAGCCTCC	(CTAT)4(CT)15	55
6	**RM85**	3	37200000	CCAAAGATGAAACCTGGATTG	GCACAAGGTGAGCAGTCC	(TGG)5(TCT)12	55
7	**RM222**	10	2600000	CTTAAATGGGCCACATGCG	CAAAGCTTCCGGCCAAAAG	(CT)18	55
8	**RM247**	12	3185384	TAGTGCCGATCGATGTAACG	CATATGGTTTTGACAAAGCG	(CT)16	55
9	**RM328**	9	3,726,000	CATAGTGGAGTATGCAGCTGC	CCTTCTCCCAGTCGTATCTG	(CAT)5	55
10	**RM337**	8	152299	GTAGGAAAGGAAGGGCAGAG	CGATAGATAGCTAGATGTGGCC	(CTT)4-19-(CTT)8	55
11	**RM340**	6	28599181	GGTAAATGGACAATCCTATGGC	GACAAATATAAGGGCAGTGTGC	(CTT)8T3(CTT)14	55
12	**RM470**	4	28,090,431	TCCTCATCGGCTTCTTCTTC	AGAACCCGTTCTACGTCACG	(CTT)14	55
13	**RM472**	1	37889084	CCATGGCCTGAGAGAGAGAG	AGCTAAATGGCCATACGGTG	(GA)21	55
14	**RM506**	8	435648-35677	CGAGCTAACTTCCGTTCTGG	GCTACTTGGGTAGCTGACCG	(CT)13	55
15	**RM1812**	11	2405106	CAGCTAGTGAGCTCCTAGTG	GCTAACCCACCAACTTATTC	(AT)16	55
16	**RM3701**	11	8100974	GAGCTAGAGGGAGGAGGTGC	TTGACTGATAGCCGATTGGG	(GA)15	55
17	**RM6947**	12	23974120	ATTAAACGTCCACTGCTGGC	GCTAGGTTAGTGGTGCAGGG	(TTC)8	55
18	**RM14978**	3	13880447	TATCTGCAGGTGCGTGTAAATGG	GCATATAGAGCGAGTAAGCGAGAGG	(TC)10	55
19	**RM18776**	5	21661718	CTCCAGGAGGGTACAAATTCTGC	CCATTGGAACATAGCAAGTGATCG	(GA)13	53
20	**RM22034**	7	26403619	CCAGTTTATCTTCTGCACCTTCTCG	TCTTTGAGCAGATGGCTAACAAGG	(ATTA)5	53
21	**RM24161**	9	12311985	GTATGGCGAGACCCTACAGACC	GACCCACTTAATGTGTCACAAGG	(TATT)5	54
22	**RM223**	8	20650060	GAGTGAGCTTGGGCTGAAAC	GAAGGCAAGTCTTGGCACTG	(CT)25	55
23	**RM440**	5	19912517	CATGCAACAACGTCACCTTC	ATGGTTGGTAGGCACCAAAG	(CTT)22	55
24	**RM201**	9	20174289	CTCGTTTATTACCTACAGTACC	CTACCTCCTTTCTAGACCGATA	(CT)17	55
25	**RM216**	10	5,352,766	GCATGGCCGATGGTAAAG	TGTATAAAACCACACGGCCA	(CT)18	55
26	**RM258**	10	18014265	TGCTGTATGTAGCTCGCACC	TGGCCTTTAAAGCTGTCGC	(GA)21(GGA)3	55

27	**RM286**	11	383711	GGCTTCATCTTTGGCGAC	CCGGATTCACGAGATAAACTC	(GA)16	55
28	**RM3735**	4	26210755	GCGACCGATCAGCTAGCTAG	ATAACTCCTCCCTTGCTGCC	(GA)16	55
29	**RM1347**	2	5314190	AACAAATTAAACTGCCAAG	GTCTTATCATCAGAACTGGA	(AG)23	55
30	**RM7571**	7	10369821	CCTTATGCCCCCTTCCTTAC	TCGTCTCATGGAGCCACC	(TCTA)6	61
31	**RM14723**	3	9,223,269	GCAAAGTCCTTTGGACAGGTAGC	CGTCCCAGATCAAAGTACACTCTTCC	(GA)29	54
32	**RM103**	6	30889151	CTTCCAATTCAGGCCGGCTGGC	CGCCACAGCTGACCATGCATGC	(GAA)5	55
33	**RM315**	1	36734135	GAGGTACTTCCTCCGTTTCAC	AGTCAGCTCACTGTGCAGTG	(AT)4(GT)10	55
34	**RM225**	6	3416533	TGCCCATATGGTCTGGATG	GAAAGTGGATCAGGAAGGC	(CT)18	55
35	**RM486**	1	34955554	CCCCCCTCTCTCTCTCTCTC	TAGCCACATCAACAGCTTGC	(CT)14	55
36	**RM256**	8	24270635	GACAGGGAGTGATTGAAGGC	GTTGATTTCGCCAAGGGC	(CT)21	55
37	**RM1113**	4	34085697	GGGCGCATGTGTATTTCTTC	TGGGGAAAAACCACAAGCC	(AG)12	55
38	**RM3423**	4	30,957,747	AGCAGGCATATAAAGGTGCC	TGGCCTCAGATTCAGGAAAC	(CT)18	50
39	**RM6100**	10	18,816,637	TCCTCTACCAGTACCGCACC	GCTGGATCACAGATCATTGC	(CGA)8	50
40	**RM590**	10	23043156	CATCTCCGCTCTCCATGC	GGAGTTGGGGTCTTGTTCG	(TCT)10	55
41	**RM5793**	7	17489638	ACTCTCTTGCGCAACTCCTC	GATAATGCTAGCTGCTGGCC		50
42	**RM405**	5	3073406	TCACACACTGACAGTCTGAC	AATGTGGCACGTGAGGTAAG	(AC)14	55
43	**RM547**	8	5591403	TAGGTTGGCAGACCTTTTCG	GTCAAGATCATCCTCGTAGCG	(ATT)19	55
44	**RM7364**	9	9561213	TTCGTGGATGGAGGGAGTAC	AGCGTTTGTAGGAGTGCCAC	(CTAT)9	50
45	**RM205**	9	22720624	CTGGTTCTGTATGGGAGCAG	CTGGCCCTTCACGTTTCAGTG	(CT)25	55
46	**RM167**	11	4073024	GATCCAGCGTGAGGAACACGT	AGTCCGACCACAAGGTGCGTTGTC	(GA)16	53
47	**RM229**	11	18,407,879	CACTCACACGAACGACTGAC	CGCAGGTTCTTGTGAAATGT	(TC)11(CT)5C3(CT)5	50
48	**RM20A**	12	970538	ATCTTGTCCCTGCAGGTCAT	GAAACAGAGGCACATTTCATTG	(ATT)14	55
49	**RM235**	12	26107904	AGAAGCTAGGGCTAACGAAC	TCACCTGGTCAGCCTCTTTC	(CT)24	55
50	**RM7003**	12	6775083	GGCAGACATACAGCTTATAGGC	TGCAAATGAACCCCTCTAGC	(AAAC)6	50
51	**RM5436**	7	9074712	CAAAGGGGGTGTCCTCTATG	GTTGCTCGTCCTACATGTGC		50
52	**RM25181**	10	8849270	AAAGAGCTTCCCTAATGGCTTCG	GAGAGAATGACCTCTCCCAAGACC	(TTC)22	55
53	**RM469**	6	564,135	AGCTGAACAAGCCCTGAAAG	GACTTGGGCAGTGTGACATG	(AG)15	55
54	**RM6547**	1	34693224	TCCATCCTTCTCCTCTCGTG	AGCCACCCCCATATATAGCC	(GCT)9	50
55	**RM152**	8	682963	GAAACCACCACACCTCACCG	CCGTAGACCTTCTTGAAGTAG	(GGC)10	55
56	**RM148**	3	35835805	ATACAACATTAGGGATGAGGCTGG	TCCTTAAAGGTGGTGCAATGCGAG	(TG)12	50

57	**RM421**	5	23976333	AGCTCAGGTGAAACATCCAC	ATCCAGAATCCATTGACCCC	(AGAT)6	55
58	**RM2634**	2	20495111	GATTGAAAATTAGAGTTTGCAC	TGCCGAGATTTAGTCAACTA	(AT)31	55
59	**RM248**	7	29,339,845	TCCTTGTGAAATCTGGTCCC	GTAGCCTAGCATGGTGCATG	(CT)25	55
60	**RM7179**	6	19728535	CACGTGTCAGCTTAAGAGCG	TTACATCATAAGCCCGCAGG	(ATAG)6	50
61	**RM215**	9	21189110	CAAAATGGAGCAGCAAGAGC	TGAGCACCTCCTTCTCTGTAG	(CT)16	55
62	**RM324**	2	11389704	CTGATTCCACACACTTGTGC	GATTCCACGTCAGGATCTTC	(CAT)21	55
63	**RM317**	4	29060978	CATACTTACCAGTTCACCGCC	CTGGAGAGTGTCAGCTAGTTGA	(GC)4(GT)18	55
64	**RM174**	2	7006085	AGCGACGCCAAGACAAGTCGGG	TCCACGTCGATCGACACGACGG	(AGG)7(GA)10	67
65	**RM556**	8	22339816	ACTCCAAACCTCACTGCACC	TAGCACACTGAACAGCTGGC	(CCAG)6	55
66	**RM257**	9	17719660	CAGTTCCGAGCAAGAGTACTC	GGATCGGACGTGGCATATG	(CT)24	55
67	**RM502**	8	26492117	GCGATCGATGGCTACGAC	ACAACCCAACAAGAAGGACG	(TG)10	55
68	**RM331**	8	12294124	GAACCAGAGGACAAAAATGC	CATCATACATTTGCAGCCAG	[(CTT)4GTT]2(CTT)11	55
69	**RM403**	1	29384585	GCTGTGCATGCAAGTTCATG	ATGGTCCTCATGTTCATGGC	(GA)8	55
70	**RM309**	12	21454591	GTAGATCACGCACCTTTCTGG	AGAAGGCCTCCGGTGAAG	(GT)13	55
71	**RM6641**	2	4633966	GGGTCTCGATTCTCAGTTGG	CAGAACCACTCATGCACACC	(GTA)14	55
72	**RM3**	6	19499320	ACACTGTAGCGGCCACTG	CCTCCACTGCTCCACATCTT	(GA)2GG(GA)25	55
73	**RM594**	1	15158295	GCCACCAGTAAAAGCAATAC	TTGATCTGCTAGTGAGACCC	(GA)n	55
74	**RM3392**	3	3825907	GTCCAATGATTCGTTCCCAC	CTTCACCGTTCACCAATTCC	(CT)17	55
75	**RM1278**	3	4561347	ATATAAAGGTGGCACGACAG	GCACTTGAACTCTAATTCTCC	(AG)17	55
76	**RM168**	3	28091534	TGCTGCTTGCCTGCTTCCTTT	GAAACGAATCAATCCACGGC	T15(GT)14	50
77	**RM3375**	1	18729953	TTGACCTCCTCCTCCACAAC	TTGCAAGGAAACTAGGAGGG	(CT)16	55
78	**RM282**	3	12407382	CTGTGTCGAAAGGCTGCAC	CAGTCCTGTGTTGCAGCAAG	(GA)15	55
79	**RM26632**	11	14702841	CCAATCACAACCCTCCATCACC	CCAAAGAGCAACATTGGTTGTGC	(TCTT)9	50
80	**RM1341**	11	19677083	AACCTGGAGGTGCTGGTCTC	TTTCTCCCCCCCAACCAC		50
81	**RM4112**	11	24646850	TGGCAAAGTCAGTAGTCCTTCCACAA	GCCATTCCCCCAACAGCTCC	(TA)14	55
82	**RM20377**	6	24,320,992	GTGTGTGATGTGCATGTTTCTGC	CATGTGATGCCCTGTAGGAACC	(CT)33	50
83	**RM210**	8	22471837	TCACATTCGGTGGCATTG	CGAGGATGGTTGTTCACTTG	(CT)23	55
84	**RM218**	3	8405368	TGGTCAAACCAAGGTCCTTC	GACATACATTCTACCCCCGG	(TC)24ACT5(GT)11	55
85	**RM494**	6	31088146	GGGAGGGGATCGAGATAGAC	TTTAACCTTCCTTCCGCTCC	(AGA)16	55
86	**RM336**	7	21,871,205	CTTACAGAGAAACGGCATCG	GCTGGTTTGTTTCAGGTTCG	(CTT)18	55

87	**RM3475**	1	26041024	GTCGGTTTGCCTAGTTGAGC	TTCCTCGGTGTATGGGTCTC	(CT)22	55
88	**RM480**	5	27,313,250	GCTCAAGCATTCTGCAGTTG	GCGCTTCTGCTTATTGGAAG	(AC)30	55
89	**RM566**	8	14704764	ACCCAACTACGATCAGCTCG	CTCCAGGAACACGCTCTTTC	(CCAG)6	55
90	**RM11701**	1	32026621	CTGGTGGAGTTGCAGTGCCTCTAGC	CCTTGCTGCTTTCTCATTGAAACTGG	(CT)18	56
91	**RM220**	1	4424392	GGAAGGTAACTGTTTCCAAC	GAAATGCTTCCCACATGTCT	(CT)17	55
92	**RM488**	1	24807508	CAGCTAGGGTTTTGAGGCTG	TAGCAACAACCAGCGTATGC	(GA)17	55
93	**RM6374**	2	15181966	TGAGGACGCTGATTGTCAAC	GCTGCCCCTATTATTTCACC	(GAA)16	55
94	**RM233**	2	2069848	CCAAATGAACCTACATGTTG	GCATTGCAGACAGCTATTGA	(CT)20	55
95	**RM112**	2	32013785	GGGAGGAGAGGCAAGCGGAGAG	AGCCGGTGCAGTGGACGGTGAC	(GAA)5	55
96	**RM13600**	2	24246249	GGTTAACCTTTCTCGCTCTTTGG	ATGATCCAAACCCACTGTCTTCC	(AG)11	50
97	**RM495**	1	215956	AATCCAAGGTGCAGAGATGG	CAACGATGACGAACACAACC	(CTG)7	55
98	**RM493**	1	12280117	TAGCTCCAACAGGATCGACC	GTACGTAAACGCGGAAGGTG	(CTT)9	55
99	**RM444**	9	5925016	GCTCCACCTGCTTAAGCATC	TGAAGACCATGTTCTGCAGG	(AT)12	55
100	**RM468**	3	32674852	CCCTTCCTTGTTGTGGCTAC	TGATTTCTGAGAGCCAACCC	(TAT)8	55
101	**RM6054**	5	22779263	CCCTCCGTACGGATACACAC	CTCTTCGGCTTCATCTCCTC	(CCG)12	55
102	**RM509**	5	16324561	TAGTGAGGGAGTGGAAACGG	ATCGTCCCCACAATCTCATC	(TC)11	55
103	**RM5638**	1	20934810	GGCTTCCTCATCGCCATC	CTGAGCAGCATTCCAGTCTG	(AAG)13	55
104	**RM8044**	7	24,195,172	AGTACTTGTCTCCTTAGCAG	CAATATTCACTCAACTCTCA	(CTT)18	55
105	**RM8271**	8	7616956-617315	TCTTGAGAAATCTGCCATTC	ACTGATGTGCATTTCGTC	(AG)32	55
106	**RM171**	10	19048795	AACGCGAGGACACGTACTTAC	ACGAGATACGTACGCCTTTG	(GATG)5	55
107	**RM16686**	4	14718643	GGCACTGCTTGCATATGGATCG	TGCCGGCGAACTTATCCTCTCC	(GGA)10	53
108	**RM434**	9	15662573	GCCTCATCCCTCTAACCCTC	CAAGAAAGATCAGTGCGTGG	(TC)12	55
109	**RM6091**	11	13405326	GCTGTCCTGTCCTTGAATCC	TGGTAGGCTGGTGACATGC	(CCT)11	50
110	**RM209**	11	17808335	ATATGAGTTGCTGTCGTGCG	CAACTTGCATCCTCCCCTCC	(CT)18	55
111	**RM245**	9	22300000	ATGCCGCCAGTGAATAGC	CTGAGAATCCAATTATCTGGGG	(CT)14	55
112	**RM1089**	5	5356127	CAGAAGGATTATCTCGATACC	AATAGGGCTTGAAATAAATTG	(AC)33	55
113	**RM228**	10	22243157	CTGGCCATTAGTCCTTGG	GCTTGCGGCTCTGCTTAC	(CA)6(GA)36	55
114	**RM401**	4	13154172	TGGAACAGATAGGGTGTAAGGG	CCGTTCACAACACTATACAAGC	(CT)15	55
115	**RM11**	7	19256914	TCTCCTCTTCCCCCGATC	ATAGCGGGCGAGGCTTAG	(GA)17	55
116	**RM3351**	5	20696671	ATGGAAGGAATGGAGGTGAG	TACCCCTACGTCGATCGATC	(CT)15	55

117	**RM5749**	4	19950587	GTGACCACATCTATATCGCTCG	ATGGCAAGGTTGGATCAGTC	(ACT)8	55
118	**RM335**	4	688353	GTACACACCCACATCGAGAAG	GCTCTATGCGAGTATCCATGG	(CTT)25	55
119	**RM144**	11	28281693	TGCCCTGGCGCAAATTTGATCC	GCTAGAGGAGATCAGATGGTAGTGCATG	(ATT)11	55
120	**RM300**	2	13191380	GCTTAAGGACTTCTGCGAACC	CAACAGCGATCCACATCATC	(GTT)14	55
121	**RM1132**	7	23984489	ATCACCTGAGAAACATCCGG	CTCCTCCCACGTCAAGGTC	(AG)12	55
122	**RM400**	6	28431560	ACACCAGGCTACCCAAACTC	CGGAGAGATCTGACATGTGG	(ATA)63	55
123	**RM471**	4	18824746	ACGCACAAGCAGATGATGAG	GGGAGAAGACGAATGTTTGC	(GA)12	55
124	**RM243**	1	7970722	GATCTGCAGACTGCAGTTGC	AGCTGCAACGATGTTGTCC	(CT)18	55
125	**RM467**	10	13488471	GGTCTCTCTCTCTCTCTCTCTCTC	CTCCTGACAATTCAACTGCG	(TC)21	55
126	**RM564**	3	18587434	CATGGCCTTGTGTATGCATC	ATGCAGAGGATTGGCTTGAG	(GT)14	55
127	**RM8007**	7	7710329	AATAGGATGGATCATGGATA	CATCTCATCAGGAACCTAAC	(AT)40	55
128	**RM441**	11	6081100	ACACCAGAGAGAGAGAGAGAGAG	TCTGCAACGGCTGATAGATG	(AG)13	55
129	**RM518**	4	2030135	CTCTTCACTCACTCACCATGG	ATCCATCTGGAGCAAGCAAC	(TC)15	55
130	**RM253**	6	5425408	TCCTTCAAGAGTGCAAAACC	GCATTGTCATGTCGAAGCC	(GA)25	55
131	**RM274**	5	26848154	CCTCGCTTATGAGAGCTTCG	CTTCTCCATCACTCCCATGG	(GA)15-7-(CGG)5	55
132	**RM242**	9	18810067	GGCCAACGTGTGTATGTCTC	TATATGCCAAGACGGATGGG	(CT)26	55
133	**RM3231**	8	3838134	AACACGAAGACCGGCCTC	CAGGTAGGAGCATGAGAGCC	(CT)12	55
134	**RM5687**	4	15742285	GATCGCTGGCGATTGATC	GACTTGTGGGGTGGTTTTTG	(AAT)17	50
135	**RM5626**	3	24864350	GCAGACGAGATGAGATCG	GTAGAGGATGGGCAGCAG	(AAG)11	55
136	**RM452**	2	9563257	CTGATCGAGAGCGTTAAGGG	GGGATCAAACCACGTTTCTG	(GTC)9	55
137	**RM14960**	3	13282361-13282553	GTCAAGACCACCGCCTGGTTCC	CGACGAGGAGGTTATGGGTCTGG	(CGA)7	
138	**RM558**	11	1608847-1609092	GAACTCCTCGAACTCGATGC	AGGCATTCAACCTGTTCGAC	(ATTG)5	55
139	**RM406**	2	35236078-35236220	GAGGGAGAAAGGTGGACATG	TGTGCTCCTTGGGAAGAAAG	(GA)17	55
140	**RM522**	1	5244852-5244994	ACCAGAGAAGCCCTCCTAGC	GTTCTGTGGTGGTCACGTTG	(AAT)6	55
141	**RM10124**	1	2309722-2310063	AGATCAGCATGGGTACATTCACTAGG	AATCAGCAGTGTTAGATGCCAAGG	(AATA)5	
142	**RM181**	11	201993-202275	ACGGGAGCTTCTCCGACAGCGC	TATGCTTTTGCCGTGTGCCGCG	(CT)13(AT)19	67
143	**RM175**	3	3865706-3865940	CTTCGGCGCCGTCATCAAGGTG	CGTTGAGCAGCGCGACGTTGAC	(CCG)8	67

Supplementary Table S3. Significant marker-trait associations detected for germination rate and early seedling growth parameters by GLM approach at p<0.01 using 143 SSR markers.

Sl.No.	Trait	Marker	Chr	Position	marker_F	marker_p	marker_Rsq
1	RSG	B-3	Unknown	5	9.28475	0.00284	0.03466
2	RSG	J-1	Unknown	27	27.20863	7.73E-07	0.0892
3	RSG	J-4	Unknown	30	7.41018	0.00745	0.02807
4	RSG	T-2	Unknown	56	15.24579	1.56E-04	0.05441
5	RSG	AG-1	Unknown	91	10.25151	0.00175	0.03799
6	RSG	AG-2	Unknown	92	7.22102	0.00823	0.02739
7	RSG	AP-1	Unknown	109	8.17726	0.005	0.03079
8	RSG	BH-1	Unknown	159	10.51694	0.00153	0.03889
9	RSG	CG-1	Unknown	221	14.93372	1.81E-04	0.05341
10	RSG	CI-3	Unknown	230	9.11	0.00311	0.03405
11	RSG	CU-3	Unknown	274	8.39526	0.00447	0.03156
12	RSG	CX-2	Unknown	282	9.68953	0.00232	0.03606
13	RSG	DC-1	Unknown	300	10.79068	0.00134	0.03982
14	RSG	EC-3	Unknown	366	14.30325	2.44E-04	0.0514
15	RSG	EL-1	Unknown	384	11.65777	8.73E-04	0.04273
16	RSG	EL-2	Unknown	385	8.54084	0.00415	0.03207
17	RGR	O-1	Unknown	44	10.65642	0.00143	0.05988
18	RGR	BD-1	Unknown	150	7.4275	0.00738	0.04279
19	RGR	CQ-1	Unknown	258	8.27259	0.00476	0.04735
20	RGR	CQ-2	Unknown	259	7.94408	0.00564	0.04559
21	RGR	CV-1	Unknown	276	9.36851	0.00272	0.05317
22	RGR	DM-1	Unknown	326	8.74447	0.00374	0.04987
23	RGR	DS-1	Unknown	340	7.02293	0.00913	0.04059
24	RGR	EK-1	Unknown	382	9.85569	0.00213	0.05572
25	AGR	B-3	Unknown	5	7.02842	0.00911	0.05096
26	AGR	G-1	Unknown	18	10.46761	0.00157	0.07389
27	AGR	J-1	Unknown	27	16.07491	1.06E-04	0.1088
28	AGR	T-2	Unknown	56	8.60253	0.00402	0.06161
29	AGR	AC-1	Unknown	82	9.1193	0.00309	0.06505
30	AGR	BH-1	Unknown	159	10.79532	0.00133	0.07601
31	AGR	CG-1	Unknown	221	7.88757	0.00581	0.0568
32	AGR	CG-2	Unknown	222	11.25419	0.00106	0.07897
33	AGR	CH-1	Unknown	225	8.39513	0.00447	0.06022
34	AGR	CY-4	Unknown	286	6.90968	0.00969	0.05014
35	AGR	DC-1	Unknown	300	10.75337	0.00136	0.07574
36	MGR	C-1	Unknown	6	6.90342	0.00973	0.02679
37	MGR	C-2	Unknown	7	7.19427	0.00834	0.02786
38	MGR	F-3	Unknown	17	12.47855	5.85E-04	0.04639

39	MGR	I-2	Unknown	26	13.82533	3.06E-04	0.05088
40	MGR	X-1	Unknown	68	8.46517	0.00432	0.03245
41	MGR	X-2	Unknown	69	15.24724	1.56E-04	0.05552
42	MGR	Z-1	Unknown	73	9.92712	0.00206	0.03763
43	MGR	AB-3	Unknown	80	31.13489	1.52E-07	0.10146
44	MGR	AB-4	Unknown	81	18.97614	2.81E-05	0.06725
45	MGR	AS-2	Unknown	120	9.58873	0.00244	0.03644
46	MGR	AY-1	Unknown	136	7.86476	0.00588	0.03029
47	MGR	BK-1	Unknown	168	14.34719	2.39E-04	0.05259
48	MGR	BK-2	Unknown	169	10.76402	0.00136	0.04054
49	MGR	BO-2	Unknown	178	12.27585	6.46E-04	0.04571
50	MGR	CC-1	Unknown	209	9.63976	0.00238	0.03662
51	MGR	CM-3	Unknown	241	11.59527	9.00E-04	0.0434
52	MGR	CN-5	Unknown	248	7.108	0.00873	0.02754
53	MGR	CO-3	Unknown	251	15.87512	1.17E-04	0.05754
54	MGR	CO-4	Unknown	252	8.89955	0.00346	0.034
55	MGR	CP-4	Unknown	257	9.2386	0.00291	0.03521
56	MGR	DC-1	Unknown	300	8.48844	0.00426	0.03254
57	MGR	DI-2	Unknown	318	7.22993	0.00819	0.02799
58	MGR	DP-2	Unknown	333	7.70705	0.00638	0.02972

Supplementary Table S4. Significant marker-trait associations detected for germination rate and early seedling growth parameters by MLM approach at p<0.01 using 143 SSR markers.

Trait	Marker	Chr	Pos	F	p	MarkerR2
RSG	B-3	Unknown	5	8.34334	0.00459	0.05847
RSG	J-1	Unknown	27	16.54241	8.55E-05	0.11592
RSG	J-4	Unknown	30	9.81468	0.00218	0.06878
RSG	T-2	Unknown	56	7.72005	0.00634	0.0541
RSG	AG-1	Unknown	91	8.83099	0.00358	0.06188
RSG	AT-3	Unknown	123	10.32616	0.00168	0.07236
RSG	BH-1	Unknown	159	7.1075	0.00873	0.04981
RSG	CG-1	Unknown	221	14.91786	1.83E-04	0.10454
RSG	DC-1	Unknown	300	8.64751	0.00393	0.0606
RSG	EL-1	Unknown	384	9.98819	0.00199	0.06999
RGR	O-1	Unknown	44	9.21126	0.00295	0.07091
RGR	BD-1	Unknown	150	7.12592	0.00865	0.05486
RGR	EK-1	Unknown	382	8.97394	0.00333	0.06909
AGR	J-1	Unknown	27	9.56844	0.00246	0.07805
AGR	Q-2	Unknown	50	7.03617	0.00907	0.0574
AGR	BH-1	Unknown	159	10.27457	0.00173	0.08381

AGR	CG-1	Unknown	221	9.96554	0.00202	0.08129
AGR	CG-2	Unknown	222	7.76612	0.00619	0.06335
AGR	DC-1	Unknown	300	11.68419	8.62E-04	0.09531
MGR	AB-3	Unknown	80	15.01011	1.75E-04	0.1052
MGR	AB-4	Unknown	81	13.40634	3.74E-04	0.09396
MGR	AT-2	Unknown	122	7.87321	0.00586	0.05518
MGR	CM-3	Unknown	241	8.75915	0.00371	0.06139
MGR	CY-5	Unknown	287	11.2439	0.00107	0.0788
MGR	DC-1	Unknown	300	6.94926	0.00949	0.04871
